# Potassium humate and cobalt enhance peanut tolerance to water stress through regulation of proline, antioxidants, and maintenance of nutrient homeostasis

**DOI:** 10.1038/s41598-023-50714-z

**Published:** 2024-01-18

**Authors:** Ayman M. S. Elshamly, Zubair Ahmad Parrey, Abdel-Rhman Z. Gaafar, Manzer H. Siddiqui, Sadam Hussain

**Affiliations:** 1 Water Studies and Research Complex, National Water Research Centre, Cairo, Egypt; 2 Plant Physiology and Biochemistry Section, Department of Botany, Aligarh Muslim University, Aligarh, 202002 India; 3 Department of Botany and Microbiology, College of Science, King Saud University, Riyadh, Saudi Arabia; 4 College of Agronomy, Key Laboratory of Crop Physio-Ecology and Tillage in Northwestern Loess Plateau, Ministry of Agriculture, Northwest A&F University, Yangling, Shaanxi China

**Keywords:** Abiotic, Drought, Plant physiology, Fertilization, Environmental impact

## Abstract

Water stress is an important factor that substantially impacts crop production. As a result, there is a need for various strategies that can mitigate these negative effects. One such strategy is the application of potassium humate (Kh) and cobalt (Co), which have been reported to enhance the resistance of crop plants. Therefore, the present experiment was designed to investigate whether the application of Kh and Co could positively affect proline, chlorophyll and mineral elements contents, and antioxidant defense systems which in turn will mitigate the negative impact of water stress under different irrigation strategies. In 2021 and 2022, an open-field experiments were conducted by using a split-plot design. The main plots were divided to represent different irrigation strategies (ST), with additional control of full irrigation requirements (ST1). Four STs were implemented, with ST1, followed by the application of 75%, 50%, and 25% irrigation strategies in ST2, ST3, and ST4 respectively, in the next irrigation, followed by the full requirements, and so on. In the subplots, peanut plants were treated with tap water (Control), Kh at 2 g l^−1^ and 3 g l^−1^, Co, Co + Kh 2 g l^−1^ and Co + Kh 3 g l^−1^. The yield was negatively affected by the implementation of ST4, despite the increase in proline contents. Furthermore, there was a decrease in relative water content, chlorophyll content, antioxidant enzymes, protein, and mineral nutrient elements. However, the application of Kh or Co showed better improvements in most of the studied parameters. It is worth noting that there was an antagonistic relationship between Co and iron/manganese, and the intensity of this relationship was found to depend on the STs implemented. The highest mineral nutrient accumulation, chlorophyll content, relative water content, protein content, oil content, seed yield, and water productivity were observed when peanut plants were treated with Kh 3 g l^−1^ + Co under the ST2 water strategy.

## Introduction

Global climate change exacerbates the water stress that negatively impacts agriculture worldwide. Nowadays, water stress is considered a major limiting factor for crop production due to the rising temperature^1,2^. Water stress leads to the reduction of water potential in plant tissues which impairs metabolic functioning and impacts plant growth and development. Water stress also leads to minimal nutrient uptake, protein denaturation, inhibition of protein synthesis and membrane disintegration of cellular organelles^3–5^. However, plants have evolved inherent mechanisms to accumulate proteins, osmoprotectants, and secondary metabolites thereby altering phytohormone signaling and regulating antioxidant defense systems that in turn confer a degree of tolerance to stress conditions. Plants also tend to enhance the synthesis of enzymatic and non-enzymatic antioxidants in response to the production of reactive oxygen species (ROS) under stress conditions^6,7^. Plants protect themselves by changing their osmo-protective and osmoregulatory systems during water stress conditions which boosts water intake and improves their cellular functioning. Although plants can resist the change in environmental factors like water stress still the plants get affected which will lead to a decrease in their yield parameters. To, ensure better growth, yield and quality of plants, several strategies have been adapted from time to time to counteract the ill effects of water stress.

One such strategy is the application of potassium humate (Kh) alone or in combination with micronutrients like cobalt to mitigate the adverse effects of water stress^8–12^. Potassium humate considered an antitranspirant substance, is one of the efficient strategies for boosting plant resilience to water stress. Kh has a direct effect on the formation of a double layer, which reduces the rate of transpiration of the leaves thereby maintaining the water status of the plant^13,14^. Kh is also used as a plant bio-stimulant and an effective natural resource with the potential to increase organic matter, regulate osmotic potential, improve nutrition status, facilitate carbohydrate transformation, promote plant growth, and increase the water use efficiency of a plant^9,15–17^. On the other hand, cobalt (Co) has a beneficial effect due to its key role in numerous enzymes involved in N_2_ fixation^18^. Furthermore, Co has a positive impact on growth, metabolism, mineral nutrient accumulation and yield and quality in plants^8,10,19,20^. However, it is quite important to elucidate the maximum allowable concentration ranges for Co in plants due to the considerations of human health, microbiome and the environment. Based on the Codex Alimentarius^21^ and Ejaz et al.^22^ the maximum limit concentration in seeds is 50 mg kg^−1^ Co. In the same context, Khan et al.^23^ indicated it was normal that the recorded Co concentrations in plants were low and ranged between 0.1 and 10 μg g^−1^ based on the dry weight of plants. On the other hand, Bennet^24^ mentioned that for normal plant growth, the tolerable limit of Co in soil was found to be 0.2–0.5 ppm. However, Mahey et al.^25^ clarified that some plant species can grow in soil containing high Co concentrations of up to 4000–10,000 ppm. In the context of human consumption, the study conducted by Simonsen et al.^26^ has found that the typical range of daily Co intake falls within the range of approximately 5 to 40 µg day^−1^. Additionally, Lukin and Zhuikov^27^ have suggested that the maximum allowable limit for Co content in cattle feed is 1 mg kg^−1^ of feed.

Peanuts (*Arachis hypogea* L.) are a protein-rich food that can help to meet the nutritional needs of the growing population. Peanuts are also a rich source of oil which has various applications in the pharmaceutical and personal care sectors. Peanuts alone cover 40% of arable land in Africa and 27.7 million hectares globally, with an average production of 1.59 tonnes per hectare^28^. In Egypt, peanuts cover over 66,000 hectares, with a total yield of 231,223 tonnes in 2019^29^. Peanut crops and other legume crops have great demand around the world. So, it becomes imperative to boost the production of peanuts which will minimize the food scarcity pressure due to the rising population.

As a result of the preceding discussion, it is evident that maintaining water balance, nutritional status and osmoprotectant pool in plants is critical to withstand challenging environmental conditions. During stress conditions, the impact of these markers is a topic of great interest. Therefore, the current study was designed to investigate the response of peanut plants under water stress as well as the role of Kh and Co application to mitigate the negative effects of water stress. The understanding of the effects of water stress on peanut plants and their responses to these adverse conditions might aid in the development of new techniques to increase the yield attributes of peanut crops subjected to water stress.

## Materials and methods

### Study site

The experiment was carried out in the growing season of 2021 and 2022 at the experimental farm of water studies and research complex station, National Water Research Center, Toshka, Egypt which is located at latitude 22° 24′ 11″ N longitude 31° 35′ 43″ E and at an altitude of 188 m.

### Meteorological, soil and irrigation water data

The monthly meteorological data for the growing seasons were obtained from the Toshka agrometeorological station located at the site of the experiment, as shown in Table 1. The region experienced an extremely dry climate with minimal annual rainfall recorded zero during the two growing seasons of 2021 and 2022 from (May–September). The mean temperature was 33.4 and 32.9 °C for the two growing seasons of 2021 and 2022 respectively. Likewise, the mean relative humidity during the two growing seasons was 17.7 and 18.2% for the 2021 and 2022 respectively. According to the guidelines of Ditzler et al.^30^, the soil profile in the experimental site was characterized by a soft to hard consistency when it was dry, and friable when moist. The main hue notation of the soils was reddish in colour. Table 2 lists the physical and chemical characteristics of the experimental soil which were estimated according to Estefan et al.^31^. The primary source of irrigation water in the area under study was groundwater extracted from a deep well. To maintain consistency throughout the experiment, it is worth noting that in the experimental site, 3 monitoring wells were drilled to monitor the groundwater level (the groundwater table was confined between 27 and 30 m). Additionally, samples of the pumped irrigation water were taken twice i.e., before sowing and after 60 DAS to determine irrigation quality parameters (Table 3).

**Table 1:** The monthly meteorological data of the growing seasons of 2021/2022 for the experimental site.

		Temperature (°C)		Relative humidity (%)		Wind speed (MS^−1^)
		Max	Min	Max	Min	
May	2021	41.5 ± 0.20	24.0 ± 0.21	23.1 ± 0.22	2.3 ± 0.22	3.4 ± 0.23
	2022	39.0 ± 0.21	21.2 ± 0.20	27.1 ± 0.23	3.5 ± 0.20	3.1 ± 0.20
June	2021	42.0 ± 0.20	25.1 ± 0.21	30.1 ± 0.20	3.4 ± 0.21	3.3 ± 0.20
	2022	41.2 ± 0.22	24.2 ± 0.22	29.1 ± 0.21	4.9 ± 0.20	3.4 ± 0.20
July	2021	43.1 ± 0.21	24.5 ± 0.20	28.8 ± 0.22	5.3 ± 0.25	2.5 ± 0.24
	2022	42.7 ± 0.20	24.3 ± 0.21	28.1 ± 0.21	3.3 ± 0.22	2.2 ± 0.24
August	2021	42.3 ± 0.21	25.4 ± 0.19	31.3 ± 0.22	5.8 ± 0.22	2.7 ± 0.22
	2022	42.5 ± 0.21	26.0 ± 0.19	33.3 ± 0.22	5.6 ± 0.22	2.6 ± 0.22
September	2021	41.5 ± 0.19	24.4 ± 0.20	37.1 ± 0.21	9.4 ± 0.21	3.2 ± 0.24
	2022	40.7 ± 0.20	26.7 ± 0.21	37.5 ± 0.22	9.3 ± 0.22	3.1 ± 0.21

The meteorological data were obtained from Toshka Agrometeorological Station, Egypt. Values are the mean of replicates ± standard errors.

*Max* maximum temperature, *Min* minimum temperature, *MS*^−*1*^ m second^−1^, and *mm* millimeter.

**Table 2:** The physicochemical properties of soil at the experimental site before the cultivation, Egypt in 2021–2022.

Parameter	Unit	Value (cm)	
		0–20	20–40
Mechanical analysis			
Sand	% by weight	89.1 ± 0.72	87.2 ± 0.72
Silt	% by weight	4.12 ± 0.70	4.27 ± 0.70
Clay	% by weight	6.78 ± 0.70	8.53 ± 0.71
Texture		Sand	Loamy sand
Chemical analysis			
pH		7.89 ± 0.70	7.66 ± 0.70
Electrical conductivity (EC) 1:1w	ds m^−1^	0.62 ± 0.74	0.40 ± 0.77
Calcium cations (Ca^2+^)	mg kg^−1^	38.0 ± 0.70	26.1 ± 0.71
Magnesium cations (Mg^2+^)	mg kg^−1^	4.86 ± 0.80	1.2 ± 0.8
Sodium cations (Na^+^)	mg kg^−1^	94.3 ± 0.72	50.6 ± 0.70
Potassium cations (K^+^)	mg kg^−1^	8.6 ± 0.71	4.69 ± 0.72
Chloride anions (Cl^−^)	mg kg^−1^	95.7 ± 0.71	67.4 ± 0.71
Sulfate anions (SO_4_^2−^)	mg kg^−1^	153.7 ± 0.70	94.1 ± 0.73
Potassium available	mg kg^−1^	133 ± 0.72	95 ± 0.70
Organic matter	% by weight	0.1 ± 2.1	0.1 ± 2.12

Each value represents the mean of replications ± standard errors.

**Table 3:** The companied values of the chemical properties irrigation water at the experimental site, during the two growing seasons 2021–2022.

Parameter	Unit	Value	Reference
pH		6.62 ± 0.71	Estefan et al.^43^
TDS	mg l^−1^	373.0 ± 0.71	
HCO_3_	mg l^−1^	154.6 ± 0.71	
Calcium cations (Ca^2+^)	mg l^−1^	49.4 ± 0.71	
Magnesium cations (Mg^2+^)	mg l^−1^	10.1 ± 0.72	
Sodium cations (Na^+^)	mg l^−1^	87.8 ± 0.71	
Potassium cations (K^+^)	mg l^−1^	4.4 ± 0.72	
Chloride anions (Cl^−^)	mg l^−1^	98.7 ± 0.71	
Sulfate anions (SO_4_^2−^)	mg l^−1^	94.8 ± 0.71	
Cobalt concentrations	mg l^−1^	nil	

Each value represents the mean of replications ± standard errors.

*TDS* total dissolved solids.

### Experimental design and treatments

As aforementioned in the introduction section, plants are often exposed to various degrees of water stress, which triggers a cascade of several responses. In evolutionary terms, initiated with stomatal closure induced by hormone homeostasis. If water stress is prolonged, the plants tend to modify their morphological traits, which leads to profound negative effects on plant growth, development, and yield^32^. Therefore, to, ensure better growth and align with the research objectives, this study intentionally followed different water stress strategies in which it was in short term and at different intensities to elucidate the response of peanut plants. Hence, the experiment was performed in a split-plot design with three replications under a drip irrigation system. The main plot was allocated for different irrigation strategies divided into four irrigation strategies as follows; **ST1:** applied 100% irrigation requirements at all growth stages, **ST2:** applied one irrigation by 75% of water requirements and followed by one irrigation with the full requirements in the next irrigation, **ST3:** applied one irrigation by 50% of water requirements and followed by one irrigation with the full requirements in the next irrigation, **ST4:** applied one irrigation with 25% of water requirements and followed by one irrigation with the full requirements in the next irrigation. The irrigation stress schemes (**ST2**, **ST3** and **ST4**) were started from the development stage till the late season while in the initial growth stage, 100% of the irrigation water requirements were applied.

To irrigate the plants, a drip irrigation system was used. The drip lateral diameter was 16 mm of P.E tube and GR drippers built-in line, with emitters spaced at 30 cm along the drip lines and 75 cm spacing between drip lines. Furthermore, there was a buffer zone between each irrigation treatment of 3 m to prevent interactions and horizontal leakage. Therefore, the buffer zone areas weren't cultivated or used throughout the experiment. Moreover, the plot was equipped with a manometer valve to control the operating pressure at 1 bar. The plots were also equipped with a flow emitter for discharge with 4.0 l h^−1^ to control the mounts of the targeted irrigation water requirements. In the subplots, the following applications were applied at tap water (control), Kh 2 g l^−1^, Kh 3 g l^−1^, Co, Kh 2 g l^−1^ + Co and Kh 3 g l^−1^ + Co. The Co was applied in the form of CoSO_4_·7H_2_O and was purchased from Sigma-Aldrich Co. In addition, the Co application was injected through the drip irrigation systems in two equal doses of 3.25 mg l^−1^, which was applied after one week and 30 days from the planting date [following the recommendations of the previous studies^12,33,34^. While Kh applications were applied as soil drenches as 2 and 3 g l^−1^ [following the recommendations of previous studies^29,35–37^ which were injected by drip irrigation systems twice after 30 and 60 days from the planting date (according to the manufacturer’s recommendations). The Kh was purchased from Central China Trading Co. Ltd (10% K_2_O). The different plant growth stages of peanut were divided into four stages according to^38^ as follow: initial stage at 0–35 days after sowing (DAS), development stage at 35–55 DAS, flowering and pod formation stage at 55–95 DAS and late-season stage at 95–115 DAS.

### Crop farming

The recommended fertilizer doses were applied according to^39^ as follows: Calcium phosphate (15.5% P_2_O_5_) was added before sowing at a rate of 480 kg ha^−1^. Ammonium nitrate (33.5% N) was added at a rate of 144.0 kg ha^−1^ divided into 6 equal doses, the first dose was added as a starter dose after 15 DAS, while the remaining doses were added at 22, 30, 40, 50 and 60 DAS. Potassium sulphate (48% K_2_O) was added at a rate of 240 kg ha^−1^ after 30 DAS. The peanut plants were sown on May 21 and May 20 of 2021 and 2022, respectively at the rate of 180 kg pods ha^−1^ (120 kg seeds ha^−1^). Two peanut seeds (Giza 6) were drilled per hill and seeded on one side of the dripper's jet after being inoculated with root nodules bacteria (*Rhizobium leguminosarum*). The seeds of peanut were purchased from the oil crops department, field crops institute and agricultural research center, Egypt. This cultivar is recommended as a high-yielding commercial cultivar. Plants were spaced 30 cm apart in each row, 60 cm between the rows and 5 cm deep in the soil. Moreover, this cultivar and the implemented methods in this study complied with international, national, and institutional guidelines and legislation.

### Calculations related to irrigation

#### *Crop evapotranspiration (*ETc*)*

The crop evapotranspiration of peanuts (ETc) was determined according to Fao Penman–Monteith^40^, as the following equation:

$$  {\text{ETc}} = \left( {{\text{ETo}} \times {\text{Kc}}} \right)  $$

Were, ETc = Crop evapotranspiration (mm). ETo = Reference evapotranspiration (mm). Kc = Crop coefficient.

The ST1 (100% of irrigation water requirements) were estimated according to the following equation:

$${\text{IR}}=\frac{{\text{ETc}}+{\text{Lr}}}{{\text{Ei}}}$$

were, IR = Irrigation water requirements (mm). ETc = Crop evapotranspiration (mm). Ei = The irrigation system efficiency % (85%)

The Ei values were calculated for the 40 cm soil depth based on^41^ as average values of 3rd, 7th, 17th and 25th irrigation events using the following equation:

$$ {\text{Ei }} = \left( {{\text{Ws}}/{\text{Wf}}} \right) \times {1}00 $$

where, Ws is the water amounts stored in the root zone (mm) and Wf is the amount of water applied to each plot (mm). Lr = The amount of leaching requirement was calculated according to^42^ as:

$$ {\text{Lr}} = {\text{ECw}}/\left( {{\text{5 ECe}}{-}{\text{ECw}}} \right) $$

Were: ECw = Electrical conductivity of irrigation water (dS m^−1^). ECe = Estimated electrical conductivity of the saturated soil extract corresponding to a 10% reduction of the maximum yield.

The appropriate timing of the irrigation event and the retention parameters such as (field capacity and wilting point) were determined by the pF-curve (soil water retention curve). Then, the allowed depletion in the available water content was adjusted between 55 and 60%, which was the critical limit for peanut development based on previous studies^43,44^. Accordingly, based on studying the soil moisture behaviour, the soil lost 40% of the available water content after 2 days. Hence, the irrigation event was set every 2 days. The applied water quantities for the other treatments were proportionally obtained from the (100% of irrigation water requirements-ST1) treatment. Moreover, the mm units were converted to (m^3^ ha^−1^) by multiplying with 10, as mentioned by Danielescu et al.^45^. Accordingly, the average total applied water quantities applied to peanut crops during the seasons of 2021 and 2022 were: 4716, 4002, 3582, and 3010 m^3^ ha^−1^ for ST1, ST2, ST3 and ST4 respectively.

#### The water productivity (WP)

Mathematically (WP) according to Elshamly^46^ and can be estimated by:

$${\text{WP}}= (\frac{{\text{Y}}}{{\text{IR}}})$$

were, WP = The water productivity (kg m^−3^). Y = The obtained yield (kg ha^−1^) and IR = The total irrigation water requirements (m^3^ ha^−1^).

#### Estimation of total chlorophyll, RWC, proline, phenolics, ROS, and antioxidant enzymes in peanut leaves

The total chlorophyll content of the leaves was measured by using a spectrophotometer according to Sadak et al.^47^. The total chlorophyll was estimated in the (100 mg) of fresh peanut leaves which were pulverized and gradually added with 10 ml of the acetone (80%) to extract the total chlorophyll in the dark for 48 h. The extractor was centrifuged at 10,000 × g for 5 min followed by the removal of debris. The absorbance of the supernatant was measured by using a spectrophotometer at 645 and 663 nm for chlorophyll a and b respectively. The average total chlorophyll content was expressed in (%) obtained as follows:

$$\mathrm{Total\,chlorophyll }(\mathrm{\%})=\mathrm{chlorophyll\,a }+\mathrm{chlorophyll\,b}$$

The RWC contents of the leaves were measured according to the method of Afzal^48^ after 65 DAS. The peanut leaves were collected from ten random plants from each experimental unit. Then the leaves were weighed and transferred to the test tube containing distilled water. The test tube was then kept in the dark at 4 °C overnight until the peanut leaves reached to a constant weight. The dry weight of the leaves was estimated after incubation of the turgid leaves at 70 °C for 24 h. The RWC contents were measured as follows:

$${\text{RWC}}=\frac{{\text{FW}}-{\text{DW}}}{{\text{TW}}-{\text{DW}}}\times 100$$

were, FW: Actual fresh weight of the leaf. DW: Dry weight of the leaf. TW: Turgid weight of the leaf.

At 65 DAS, peanut leaves from the top of the plant were randomly collected from ten different plants in each experimental unit. Then, the proline content was estimated using the leaves of the plant as described by Sahin^49^. The 0.5 g of fresh leaf samples were ground in liquid nitrogen with a mortar and pestle. Then 10 ml of 3% sulfosalicylic acid was subsequently added and the solution was then homogenized in a water bath at 99 °C and the solution was stored in the refrigerator until the extraction process. The supernatant was obtained by using a centrifuge at 12,000 × g for 10 min. 2 ml of supernatant was taken and mixed with both (2 ml of glacial acetic acid and 3 ml of 2.5% ninhydrin reagent). For 60 min the reaction mixture was incubated in boiling water, then it was extracted by adding 4 ml of toluene. The absorbance of the proline content was measured using a spectrophotometer at 520 nm and was repeated three times. To determine the proline content the following formula was used:

$$  {\text{Proline}}\;{\text{content}} = \frac{{{\text{Toluene}}\;\left( {{\text{ml}}} \right) \times {\text{proline}}\;({\text{mg}}/{\text{ml}})}}{{115.13\;{\text{ml}}/{\text{mol}}}} \times \frac{{{\text{samples}}\;({\text{g}})}}{5}  $$

These steps were replicated three times, and the mean value of proline contents was expressed as μ mol g^−1^ FW.

The phenolics content was analyzed according to Abu-Sree et al.^50^. Random ten peanut leaves were collected from ten different plants from each experimental unit and dried by using a hot-air oven at 40 ± 3 °C for 36 h. Then crashed and the powder was passed through a sieve mesh cloth. By using aqueous methanol (80%), the leaf powder was extracted and then evaporated by using a rotary evaporator. By mixing 0.2 ml of extracted solution with (1 ml) of the diluted Folin-Ciocalteu’s phenol reagent. After that, 0.8 ml sodium carbonate (7.5%) was added. The mixture was left at room temperature for about 30 min and then the absorbance was estimated using a UV spectrophotometer at wavelength 760 nm to measure the total phenolic content, which was expressed as mg gallic acid equivalent (GAE)/100 g of the crude extract against the blank, and the previous steps were replicated three times.

The determination of ROS as hydrogen peroxide (H_2_O_2_) and the antioxidant enzymes activities such as catalase (CAT), peroxidase (POD), superoxide dismutase (SOD) and ascorbate peroxidase (APX) in leaves was estimated according to the method of Dawood et al.^51^ and Azevedo Neto et al.^52^. The three replicates for each treatment were used to confirm the results. The peanut leaves were randomly collected from ten different plants from each experimental unit. Then, for the detection of H_2_O_2_ content, 0.5 g of peanut leaves were mixed with KH_2_PO_4_-KOH buffer (pH 7.8) to prepare the extract. Then using the TiCl_2,_ the optical density was measured by using a spectrophotometer at 410 nm and the results were finally expressed in μ mol g^−1^ FW.

The CAT enzyme activity was determined by preparing a leaf extract from 1 g of fresh peanut leaves with 4 ml of ice-cold extraction buffer consisting of [100 mM phosphate buffer (pH 7.0) + 0.1 mM EDTA + 25 mM H_2_O_2_.]. Total CAT enzyme activity was estimated by mixing 100 ml of the supernatant with (2.0 ml) of the reaction mixture consisting of 100 mM phosphate buffer (pH 7.0) + 0.1 mM EDTA + 25 mM H_2_O_2_. The CAT enzyme activity was measured by monitoring the degradation of H_2_O_2_ in the range of 1 min at 240 nm, and the results were expressed in μ mol g^−1^ FW. For the determination of POD enzyme activity, the reaction mixture of 50 ml was added with 3 ml phosphate buffer (0.1 M, pH 7.0) followed by the addition of 30 ml H_2_O_2_ (20 mM) and 50 ml guaiacol (20 mM). Then the mixture was kept in a cuvette for 10 min at room temperature, after that the optical density was estimated at 436 nm and the results were finally expressed in μ mol g^−1^ FW.

To estimate the SOD enzyme activity in peanut leaves, 1 g of fresh leaves was homogenized with K-phosphate buffer pH 6.8 and 0.1 mm EDTA for 5 min. Then the mixture was filtrated and centrifuged at 16,000 × g for 15 min. After that 50 µl of the enzymatic extract was applied to [13 mm l-methionine + 75 µm nitro blue tetrazolium chloride (NBT) + 100 µm EDTA + 2 µm riboflavin in a 50 mm potassium phosphate buffer pH 7.8]. Then the reaction was initiated by turning on the UV lamp and finally, the SOD enzyme activity was measured at a wavelength of 560 nm, and the results were expressed in μ mol g^−1^ FW.

The APX enzyme activity was measured at 290 nm for 1 min. Using 1.95 ml of the reaction mixture that contained (50 mM phosphate buffer (pH 6.0) + 0.1 mM EDTA + 0.5 mM ascorbate + 1.0 mM H_2_O_2_ + 65 ml enzyme extract). The reaction was started by monitoring the degradation of H_2_O_2_ in the range of 1 min at 290 nm, and the results were finally expressed in μ mol g^−1^ FW.

#### Determination of soil pH

Three soil samples were taken from a depth of 0–40 cm. After that, the soil samples were air-dried and then the soil was passed through a sieve. The mean of the samples was then used to determine the soil pH by a potentiometric method using an Orion 501 digital Ionalyzer research multifunctional pH meter and the soil: water ratio was found to be 2.5:1.

#### Estimation of mineral nutrient content, carbohydrates, protein and oil content

At harvest, the dried seeds of ten random peanuts from each experimental unit were weighed and ground into a fine powder. The macronutrients such as N, P, K and Ca and micronutrients like Fe, Zn and Mn were estimated according to^53–55^. Moreover, the dried leaves and roots of peanuts were weighed and ground into a fine powder to measure K contents using a flame photometer as stated by Estefan et al.^31^. And the previous steps were replicated three times.

The samples of ten different peanut plants (leaves and roots) were collected from Co-treated plots. First, the samples were dried and then crushed into a fine powder. Thereafter, the Co content was determined by using flame atomic absorption spectroscopy in an acetylene-air flame as demonstrated by Wojcieszek and Ruzik^56^. The previous steps were replicated three times, and the mean value of Co contents was expressed as mg kg^−1^. The total carbohydrates (mg g^−1^) in the peanut seeds were determined by the method described by El-Katony et al.^57^. The peanut seeds were harvested from ten random plants from each experimental unit, and replicated three times. The 0.5 g of the seeds were ground into a powder and then boiled with 5 ml of 80% ethanol. Thereafter, the mixture was centrifuged for 10 min followed by the extraction. The aliquots were then thoroughly blended with anthrone reagent (8.6 mM anthrone in 80% v/v H_2_SO_4_). The mixture was then boiled on a water bath at 80 °C for 10 min followed by cooling for 30 min on the ice. Finally, at 623 nm, the absorbance was measured by using a standard calibration curve of glucose.

To determine the protein content and oil content of the peanut seeds, ten seeds were harvested from ten random plants from each experimental unit and replicated three times. The protein content (%) was measured by multiplying the content of nitrogen (N) in seeds (%) with a coefficient of 6.25 by adopting the method of^58^. The oil content of the seeds was estimated by the following formula as described by^59^:

$$\mathrm{Oil\%}=\frac{\mathrm{Final\,weight\,of\,grains}-\mathrm{Initial\,weight\,of\,grains}}{\mathrm{Total\, sample\,weight}}\times 100$$

#### Crop measurements

The peanut crop was harvested at full maturity. The peanut yield was determined for each plot and then converted to Kg ha^−1^.

### Statistical analysis

Means of variance (ANOVA) were analyzed in all obtained data to determine any statistically significant differences using the CoStat software program CoStat^60^. The means were separated through a revised least significant difference test at the 0.05 level as per Casella^61^.

### Ethics approval and consent to participate

This manuscript is an original paper and has not been published in other journals. The authors agreed to keep the copyright rule.

## Results

### The impact of the various STs, Co and Kh on:

#### Total chlorophyll

**Figure 1:**
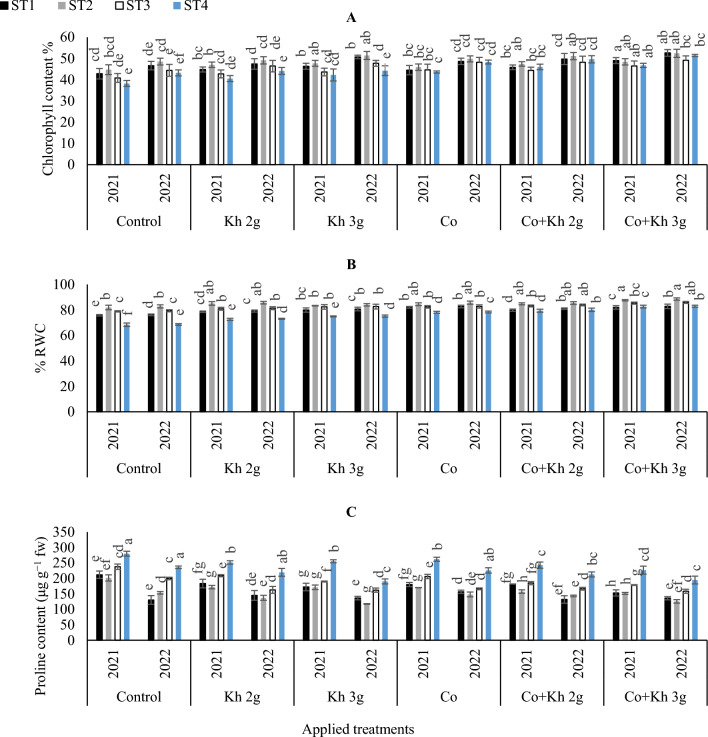
The impacts of implementing different water requirements strategies (STs) and applied cobalt applications combined with ground drench applications of Kh on the (**A)** Chlorophyll content, (**B)** RWC, and (**C)** Proline contents in the peanut leaves at two seasons of 2021/2022. Data represent the mean of three replicates of both the years of 2021 and 2022. Error bars indicate standard errors from the mean. Bars with different letters are statistically significant at *p* ≤ 0.05. Abbreviations: Control, spray with pure water; Kh (applying soil application of potassium humate at two rates 2 and 3 g l^−1^); and Co (applied two equal doses of cobalt by (3.25 mg l^−1^ for the dose), injection in the irrigation water).

The effects of various STs, Co, and Kh on the total chlorophyll content are presented in (Fig. 1A). There was a significant difference in the total chlorophyll content under various STs as compared to the control treatment as demonstrated in Tables 4 and 5. ST4 led to a decrease in total chlorophyll content under the control treatment. However, the adoption of ST2 and Kh 3 g l^−1^ applications alone or in combination with Co increased the total chlorophyll content. The highest chlorophyll content was observed under ST2 and the application of Co + Kh 3 g l^−1^. Relative to control ST2, chlorophyll content of the Co + Kh 3 g l^−1^ under ST2 were increased in the first and second season, respectively.

**Table 4:** Variance analysis of the investigated parameters in the first growing season.

Source of variation	df	CHL	RWC	PROL	SOD	POD	CAT	APX
Irrigation schemes (ST)	3	*	*	*	*	*	*	*
Examined treatments (ET)	5	*	*	*	*	*	*	*
ST × ET	15	*	*	NS	*	*	*	*
	*p*-value							
		0.001	< 0.001	< 0.001	< 0.001	< 0.001	< 0.001	< 0.001
		< 0.001	< 0.001	< 0.001	< 0.001	< 0.001	< 0.001	< 0.001
		0.003	< 0.001	0.082	< 0.001	< 0.001	< 0.001	0.027

		H_2_O_2_	PHE	N	P	Ca	KL	KR
Irrigation schemes (ST)	3	*	*	*	*	*	*	*
Examined treatments (ET)	5	*	*	*	*	*	*	*
ST × ET	15	*	*	*	*	*	*	*
	*p*-value							
		< 0.001	< 0.001	< 0.001	0.001	< 0.001	< 0.001	< 0.001
		< 0.001	< 0.001	< 0.001	0.001	< 0.001	< 0.001	< 0.001
		< 0.001	< 0.001	< 0.001	0.001	< 0.001	0.002	< 0.001

		KS	Y	WP	Fe	Mn	Zn	COL
Irrigation schemes (ST)	3	*	*	*	*	*	*	*
Examined treatments (ET)	5	*	*	*	*	*	*	*
ST × ET	15	NS	*	*	*	*	*	*
	*p*-value							
		< 0.001	< 0.001	< 0.001	< 0.001	< 0.001	< 0.001	0.002
		< 0.001	< 0.001	< 0.001	< 0.001	< 0.001	< 0.001	< 0.001
		0.007	< 0.001	0.001	0.025	< 0.001	< 0.001	< 0.001

		COR	CAR	RL	pH	OIL	PROT	
Irrigation schemes (ST)	3	*	NS	*	NS	*	*	
Examined treatments (ET)	5	*	NS	*	*	*	*	
ST × ET	15	*	NS	*	NS	*	*	
	*p*-value							
		< 0.001	0.151	< 0.001	0.34	< 0.001	< 0.001	
		< 0.001	0.372	< 0.001	0.001	< 0.001	< 0.001	
		< 0.001	0.254	0.007	0.09	< 0.001	< 0.001	

*CHL* total chlorophyll; *RWC* relative water content; *PROL* proline; *SOD* superoxide dismutase; *POD* peroxidase; *CAT* catalase; *APX* ascorbate peroxidase; *H*_*2*_*O*_*2*_ hydrogen peroxide; *PHE* phenolics; *N* nitrogen; *P* phosphorus; *Ca* calcium; *KL* potassium contents in leaves; *KR* potassium contents in roots; *KS* potassium contents in seeds; *Y* peanuts yield; *WP* water productivity; *Fe* iron; *Mn* manganese; *Zn* zinc; *COL* cobalt in leaves; *COR* cobalt in roots; *CAR* carbohydrates; *RL* root length; *pH* power of hydrogen; *PROT* protein ; *NS* non-significance.

*significance at *P* ≤ 0.05.

**Table 5:** Variance analysis of the investigated parameters in the second growing season.

Source of variation	df	CHL	RWC	PROL	SOD	POD	CAT	APX
Irrigation schemes (ST)	3	*	*	*	*	*	*	*
Examined treatments (ET)	5	*	*	*	*	*	*	*
ST × ET	15	*	*	*	*	*	*	NS
	*p*-value							
		< 0.001	< 0.001	< 0.001	< 0.001	< 0.001	< 0.001	< 0.001
		< 0.001	< 0.001	< 0.001	< 0.001	< 0.001	< 0.001	< 0.001
		0.001	< 0.001	< 0.001	< 0.001	< 0.001	< 0.001	0.070

		H_2_O_2_	PHE	N	P	Ca	KL	KR
Irrigation schemes (ST)	3	*	*	*	*	*	*	*
Examined treatments (ET)	5	*	*	*	*	*	*	*
ST × ET	15	*	*	*	*	*	*	*
	*p*-value							
		< 0.001	< 0.001	< 0.001	< 0.001	< 0.001	< 0.001	< 0.001
		< 0.001	< 0.001	< 0.001	< 0.001	< 0.001	< 0.001	< 0.001
		< 0.001	< 0.001	< 0.001	< 0.001	< 0.001	0.006	< 0.001

		KS	Y	WP	Fe	Mn	Zn	COL
Irrigation schemes (ST)	3	*	*	*	*	*	*	*
Examined treatments (ET)	5	*	*	*	*	*	*	*
ST × ET	15	NS	*	*	*	*	*	*
	*p*-value							
		< 0.001	< 0.001	< 0.001	< 0.001	< 0.001	< 0.001	0.001
		< 0.001	< 0.001	< 0.001	< 0.001	< 0.001	< 0.001	< 0.001
		0.080	< 0.001	< 0.001	0.002	< 0.001	< 0.001	< 0.001

		COR	CAR	RL	pH	OIL	PROT	
Irrigation schemes (ST)	3	*	*	*	*	*	*	
Examined treatments (ET)	5	*	*	*	*	*	*	
ST × ET	15	*	NS	*	*	*	NS	
	*p*-value							
		< 0.001	0.010	0.001	0.001	< 0.001	< 0.001	
		< 0.001	0.009	< 0.001	0.001	< 0.001	< 0.001	
		< 0.001	0.513	0.001	0.023	0.003	0.378	

*CHL* total chlorophyll; *RWC* relative water content; *PROL* proline; *SOD* superoxide dismutase; *POD* peroxidase; *CAT* catalase; *APX* ascorbate peroxidase; *H*_*2*_*O*_*2*_ hydrogen peroxide; *PHE* phenolics; *N* nitrogen; *P* phosphorus; *Ca* calcium; *KL* potassium contents in leaves; *KR* potassium contents in roots; *KS* potassium contents in seeds; *Y* peanuts yield; *WP* water productivity; *Fe* iron; *Mn* manganese; *Zn* zinc; *COL* cobalt in leaves; *COR* cobalt in roots; *CAR* carbohydrates; *RL* root length; *pH* power of hydrogen; *PROT* protein ; *NS* non-significance.

*significance at *P* ≤ 0.05.

#### RWC

The results indicated that ST4 under control treatment resulted in significantly lower values for RWC as seen in (Fig. 1B). The maximum increase in RWC was observed when Co + Kh 3 g l^−1^ was adopted under ST2.

#### Proline regulation

Proline contents were increased under ST4 and in the first season than the second, as shown in (Fig. 1C). Moreover, the highest values were obtained under the control treatment. In contrast, the lowest proline contents were observed when ST2 was adopted in combination with Co + Kh 3 g l^−1^. Relative to control ST2, the proline content of Co + Kh 3 g l^−1^ under ST2 were decreased in the first and second seasons, respectively.

#### The activities of antioxidant enzymes (SOD, POD, CAT and APX)

**Figure 2:**
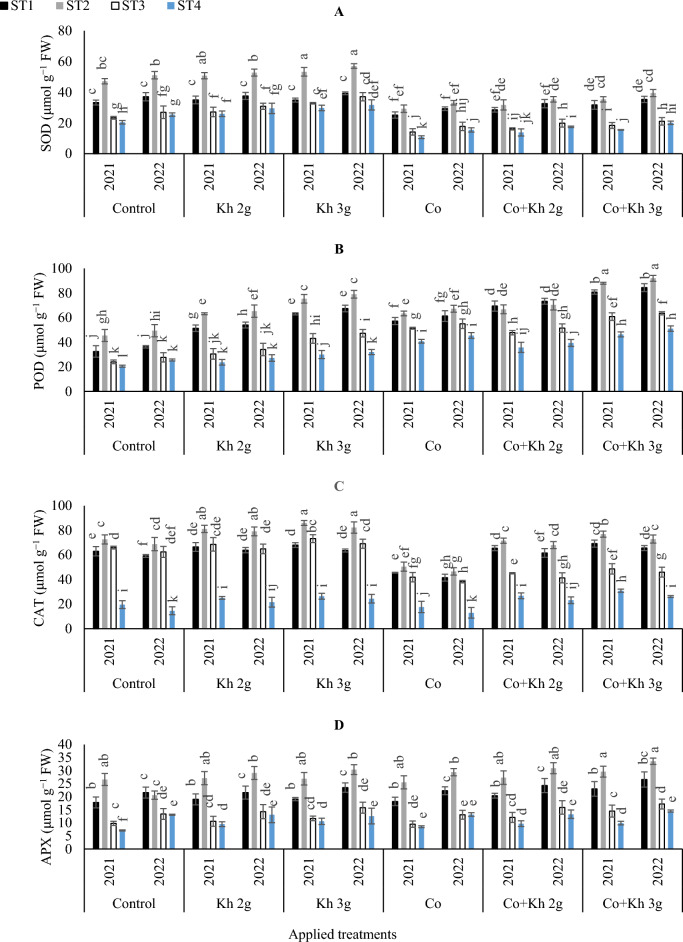
The impacts of implementing different water requirements strategies (STs) and applied cobalt applications combined with ground drench applications of Kh on the (**A**) SOD, (**B**) POD, (**C**) CAT, and (**D**) APX contents in the peanut leaves at two seasons of 2021/2022. Data represent the mean of three replicates of both the years of 2021 and 2022. Error bars indicate standard errors from the mean. Bars with different letters are statistically significant at *p* ≤ 0.05. Abbreviations: Control, spray with pure water; Kh (applying soil application of potassium humate at two rates 2 and 3 g l^−1^); and Co (applied two equal doses of cobalt by (3.25 mg l^−1^ for the dose), injection in the irrigation water).

Both Co application and increase water stress intensity led to lower SOD accumulation, as shown in (Fig. 2A). Under the control treatment in the first and second seasons, SOD content increased by adopting ST1 or ST2 as compared to the ST4. Moreover, supplying Co + Kh 2 g l^−1^ or Kh 3 g l^−1^ led to improvements in SOD under various ST schemes as compared to the Co application. Overall, the highest SOD contents were recorded using the Kh 3 g l^−1^ × ST2. While the lowest SOD content was recorded under the Co × ST4 scheme. As illustrated in (Fig. 2B), adopting the ST2 and the application of Co + Kh 3 g l^−1^ significantly achieved the highest POD. While the lowest POD contents were recorded under the control treatment of the ST4 scheme in the first season. And under (control ST4, Kh 2 g l^−1^ × ST4, and Kh 3 g l^−1^ × ST4) in the second season. On the other hand, the maximum CAT contents were observed by adopting the solitary applications of Kh 3 g l^−1^ or Kh 3 g l^−1^ under the ST2 (Fig. 2C). The lowest CAT values were observed with Co application under the ST4 in the first season. And under the control ST4 or control Co × ST4 in the second season. As shown in (Fig. 2D), the ST2 showed the highest APX content under sole or combined applications of Co + Kh. On the other hand, the lowest APX contents were recorded under control ST4 in the first season. And at the ST4 water strategy under sole or combined applications of Co + Kh, in the second season.

#### *H*_*2*_*O*_*2*_* and phenolic contents*

**Figure 3:**
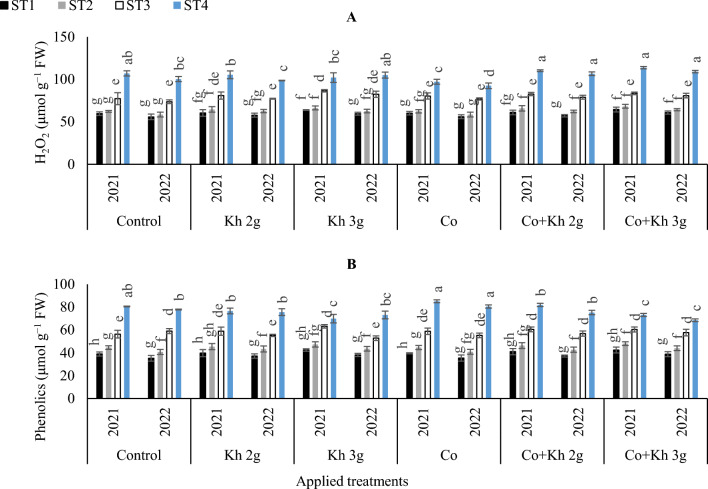
The impacts of implementing different water requirements strategies (STs) and applied cobalt applications combined with ground drench applications of Kh on the (**A**) H_2_O_2_ content, and (**B**) Phenolic contents in the peanut leaves at two seasons of 2021/2022. Data represent the mean of three replicates of both the years of 2021 and 2022. Error bars indicate standard errors from the mean. Bars with different letters are statistically significant at *p* ≤ 0.05. Abbreviations: Control, spray with pure water; Kh (applying soil application of potassium humate at two rates 2 and 3 g l^−1^); and Co (applied two equal doses of cobalt by (3.25 mg l^−1^ for the dose), injection in the irrigation water).

The highest H_2_O_2_ level was observed under control ST4 in the first and second seasons (Fig. 3A). Although that was equalled with the adoption of Co + Kh 2 g l^−1^ × ST4 or the adoptions of Co + Kh 3 g l^−1^ × ST4. The lowest H_2_O_2_ content in the first season was registered under control (ST1 and ST2). However, that was equalled when the sole application of Co under (ST1 and ST2) or the combined application of Co + Kh 2 g l^−1^ under the ST1 scheme was adopted. Likewise, in the second season, the adoptions of control (ST1 and ST2) significantly matched with the sole application of (Co, Kh 2 g l^−1^, and Kh 3 g l^−1^) in showing the lowest H_2_O_2_ content. However, that was equalled when the combined applications of Co + Kh 2 g l^−1^ under the ST1 were adopted.

The highest phenolic content was obtained with the sole application of Co under the ST4, although that significantly matched the adoption of control ST4 in the first season. While in the second season, the highest phenolic content was obtained with the sole application of Co under the ST4 scheme. On the other hand, the lowest phenolic content was observed when the ST1 was applied in the first and second seasons (Fig. 3B).

#### Maintaining the nutrient uptake homeostasis of N, P, and Ca

**Figure 4:**
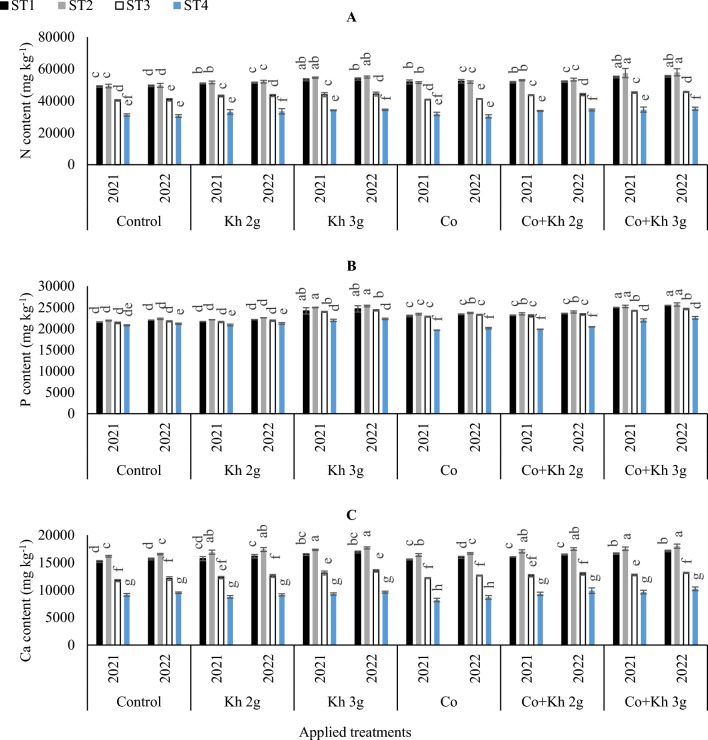
The impacts of implementing different water requirements strategies (STs) and applied cobalt applications combined with ground drench applications of Kh on the (**A**) Nitrogen(N), (**B**) Phosphorus (P), and (**C**) Calcium (Ca) in the peanut leaves at two seasons of 2021/2022. Data represent the mean of three replicates of both the years of 2021 and 2022. Error bars indicate standard errors from the mean. Bars with different letters are statistically significant at *p* ≤ 0.05. Abbreviations: Control, spray with pure water; Kh (applying soil application of potassium humate at two rates 2 and 3 g l^−1^); and Co (applied two equal doses of cobalt by (3.25 mg l^−1^ for the dose), injection in the irrigation water).

The effects of different STs, Co, and Kh on the uptake of N, phosphorus (P), and calcium (Ca) in peanuts are presented in (Fig. 4A,B and C). The lowest N uptake was observed under ST4 with the sole application of Co. Although that significantly matched the adoption of control ST4 in the first and second seasons. While the highest N uptake was observed under ST1 and ST2 with the combined application of Co + Kh 3 g l^−1^. However, that significantly matched the adoption of the sole application of Kh 3 g l^−1^ under (ST1 and ST2) in the first season. Likewise, the highest N uptake was observed under (ST1 and ST2) with the combined application of Co + Kh 3 g l^−1^. However, that significantly matched the adoption of the sole application of Kh 3 g l^−1^ under ST2 in the second season.

In terms of P uptake, the lowest values were observed by applying combined applications of Co + Kh 2 g l^−1^ or the sole application of Co under ST4, in the first and second seasons. The highest P uptake was observed with the sole Kh 3 g l^−1^ applications or both combined applications of Co + Kh 2 g l^−1^ and Co + Kh 3 g l^−1^ under ST1 or ST2 in the first and second seasons.

The highest Ca uptake was observed with the sole application of Kh 2 g l^−1^ and Kh 3 g l^−1^ under ST2. However, that significantly matched the adoption of Co + Kh 2 g l^−1^ and Co + Kh 3 g l^−1^ under the ST2 in the first and second seasons. The lowest Ca uptake was observed under the adoption of ST4 with the sole application of Co in the first and second seasons.

#### Maintaining the nutrient uptake homeostasis of K contents in peanut (leaves, roots, and seeds)

**Figure 5:**
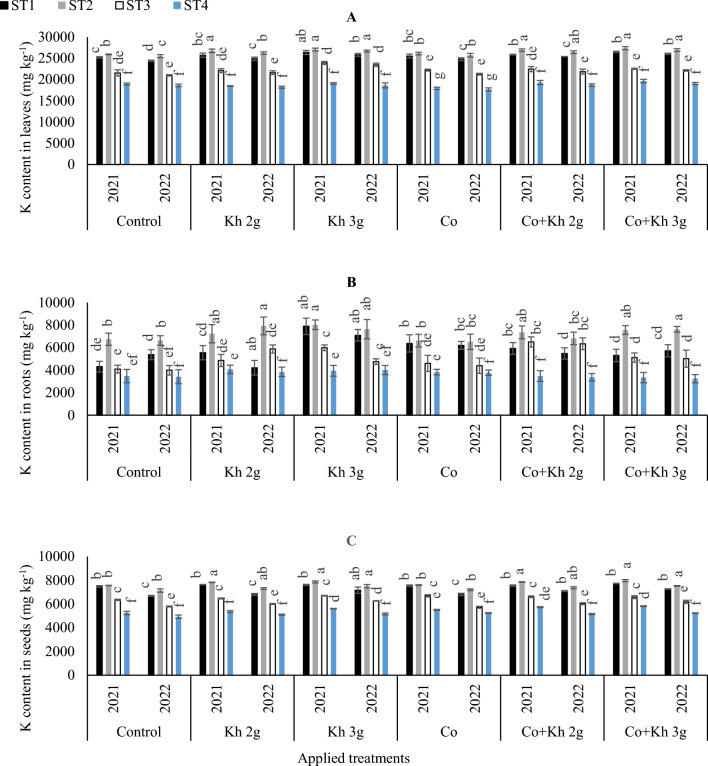
The impacts of implementing different water requirements strategies (STs) and applied cobalt applications combined with ground drench applications of Kh on the Potassium contents (K) (**A**) in peanut leaves, (**B**) in peanut roots, and (**C**) in peanut seeds at two seasons of 2021/2022. Data represent the mean of three replicates of both the years of 2021 and 2022. Error bars indicate standard errors from the mean. Bars with different letters are statistically significant at *p* ≤ 0.05. Abbreviations: Control, spray with pure water; Kh (applying soil application of potassium humate at two rates 2 and 3 g l^−1^); and Co (applied two equal doses of cobalt by (3.25 mg l^−1^ for the dose), injection in the irrigation water).

The obtained results indicated that K has a vital role can mitigating the serious repercussions to peanut plants under stressful water conditions by enhancing plant tolerance (Fig. 5A). By comparing the control treatment, it was found that the adoption of various applications had a significant difference in K contents in leaves especially under ST1 or ST2 in both seasons. In the first season, the adoptions of Kh 2 g l^−1^ under ST2 or Kh 3 g l^−1^ under (ST1 and ST2) significantly matched with the combined applications of Co + Kh 2 g l^−1^ and Co + Kh 2 g l^−1^ under the ST2 by showing the highest K contents in leaves. Likewise in the second season, the adoptions of Kh 3 g l^−1^ under ST2 significantly matched with the combined applications of Co + Kh 2 g l^−1^ and Co + Kh 2 g l^−1^ under the ST2 by showing the highest K contents in leaves. On the other hand, the lowest K content in leaves was observed with the adoptions of the sole application of Co under control ST4 in the first and second seasons.

According to the results presented in (Fig. 5B), it can be observed that the K contents in roots were higher under the extensive irrigation schemes (ST1 and ST2) compared to the (ST3 and ST4) schemes. The highest K contents in roots were obtained when Kh 3 g l^−1^ was applied with the (ST1 and ST2) schemes. However, that significantly matched the adoption of Kh 2 g l^−1^ under ST2 or the adoption of Co + Kh 2 g l^−1^ and Co + Kh 3 g l^−1^ under ST2 in the first season. Likewise, in the second season highest K contents in roots were obtained by adopting the solitary application of Kh 3 g l^−1^ under ST1 and ST2. Although that significantly matched the adoption of Kh 2 g l^−1^ under ST2, or the adoption of Co + Kh 3 g l^−1^ under ST2 in the second season. On the other side, a significant reduction in K contents in roots was observed in the control treatment where ST4 was adopted or with the adoption of Co + Kh 2 g l^−1^ and Co + Kh 3 g l^−1^ under ST4. Regarding the K contents in the seeds (Fig. 5C), the adoption of the ST2 with Kh 2 g l^−1^ and Kh 3 g l^−1^ applications matched the combined applications of Co + Kh 2 g l^−1^ and Co + Kh 3 g l^−1^ under the ST2 for attaining the highest K uptake in both seasons. On the other hand, the lowest K content in seeds in the first season was observed under control ST4 or with the sole application of Kh 2 g l^−1^ under the ST4. The lowest K content in seeds in the second season was observed under control ST4 under various sole or combined applications.

#### Maintaining the nutrient uptake homeostasis of Fe, Mn and Zn

**Figure 6:**
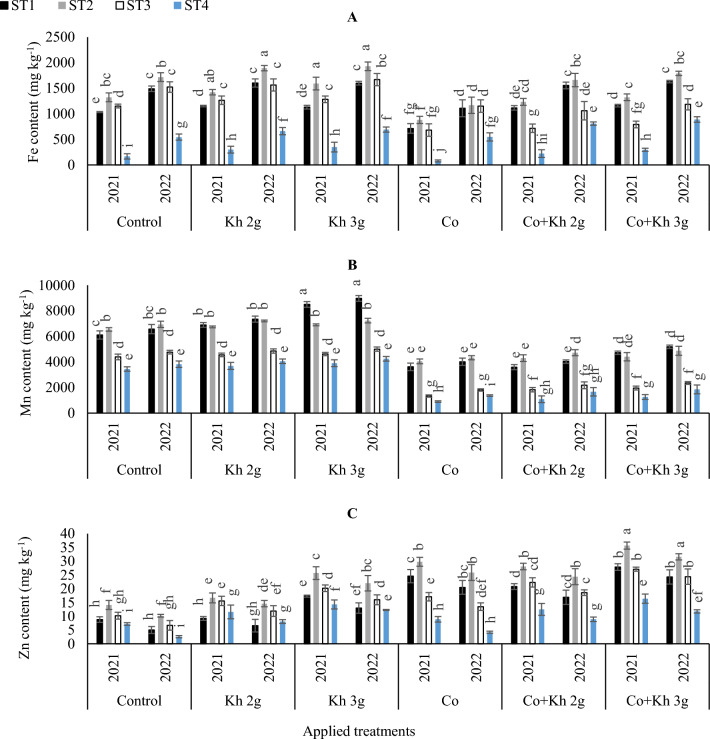
The impacts of implementing different water requirements strategies (STs) and applied cobalt applications combined with ground drench applications of Kh on the (**A**) Iron (Fe), (**B**) Manganese (Mn), and (**C**) Zinc (Zn) in the peanut seeds at two seasons of 2021/2022. Data represent the mean of three replicates of both the years of 2021 and 2022. Error bars indicate standard errors from the mean. Bars with different letters are statistically significant at *p* ≤ 0.05. Abbreviations: Control, spray with pure water; Kh (applying soil application of potassium humate at two rates 2 and 3 g l^−1^); and Co (applied two equal doses of cobalt by (3.25 mg l^−1^ for the dose), injection in the irrigation water).

It was observed that an increase in water stress intensity leads to a decrease in the accumulation of micronutrients (Fig. 6A). Based on the obtained results in this study, under the control treatment, Fe content increased with the adoption of high irrigation quantities as compared to the low irrigation scheme. The adoption of supplying Co as a sole or combined application led to the lowest values of Fe content in peanut seeds under various ST schemes. However, the application of Kh showed the highest Fe accumulation under the Kh 2 g l^−1^ × ST2 or Kh 3 g l^−1^ × ST2, in the first and second seasons. The highest Mn content was obtained with the sole application of Kh 3 g l^−1^ under ST1, in the first and second seasons (Fig. 6B). Whereas in the first season, the lowest Mn concentration was observed with the sole application of Co under ST4. Although that significantly matched the adoption of the combined applications of Co + Kh 2 g l^−1^ under the ST4 scheme. In the second season, the lowest Mn content was observed with the sole application of Co under the ST4 scheme. The highest Zn accumulation was noted with the combined application of Co and Kh 3 g l^−1^ under the ST2 in the first and second seasons. Whereas the lowest Zn content was observed in the control treatment under ST4 in the first and second seasons (Fig. 6C).

#### Maintaining the nutrient uptake homeostasis of Co contents in peanut (leaves and roots)

**Figure 7:**
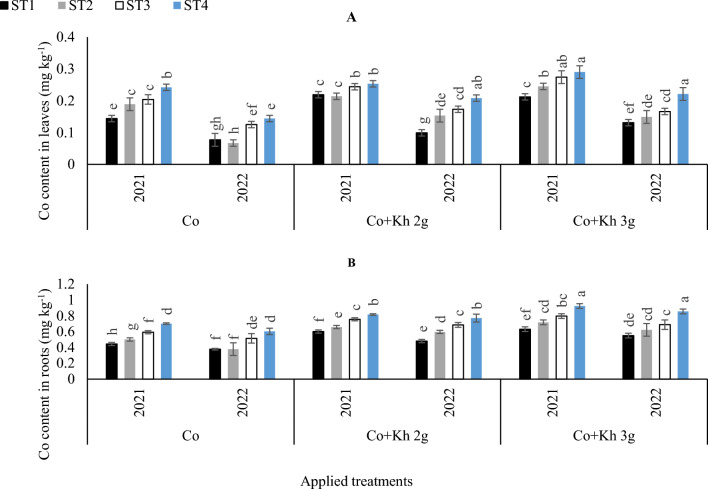
The impacts of implementing different water requirements strategies (STs) and applied cobalt applications combined with ground drench applications of Kh on the (**A**) Cobalt contents (Co) in peanut leaves and (**B**) Cobalt contents (Co) in peanut roots at two seasons of 2021/2022. Data represent the mean of three replicates of both the years of 2021 and 2022. Error bars indicate standard errors from the mean. Bars with different letters are statistically significant at *p* ≤ 0.05. Abbreviations: Control, spray with pure water; Kh (applying soil application of potassium humate at two rates 2 and 3 g l^−1^); and Co (applied two equal doses of cobalt by (3.25 mg l^−1^ for the dose), injection in the irrigation water).

In contrast to the full irrigation strategy (ST1), irrigated peanut plants with the stressful irrigation schemes showed the highest accumulations of Co contents in the leaves, as can be seen in (Fig. 7A). Based on the obtained findings, the accumulations of Co contents in the leaves attained lower values in the second season than the first. Moreover, it was observed that the combined applications of Co + Kh 2 g l^−1^ attained higher Co contents in peanut leaves by adopting ST3 and ST4 in the first season. The same result was observed by adopting ST4 and applying Co + Kh 2 g l^−1^ or Co + Kh 3 g l^−1^ in the second season. Conversely, it was shown that the lowest contents of Co could be attained by applying solitary applications of Co under the ST1 water scheme (0.144 mg kg^−1^), in the first season. In the second season, there were insignificant variations among the solitary Co × ST1 or Co × ST2 in attaining the lowest contents of Co in peanut leaves. On the other hand, the highest Co contents in peanut roots were achieved through the adoption of Co + Kh 3 g l^−1^ under the ST4 scheme, in the first and second seasons. The lowest Co contents in peanut roots were obtained through the solitary application of Co under ST1 in the first season or by applying Co × (ST1 and ST2) in the second season (Fig. 7B). The highest Co contents in peanut roots were recorded with the combined application of Co + Kh 3 g l^−1^ under the ST4, in the first and second seasons.

#### Soil pH and root length

**Figure 8:**
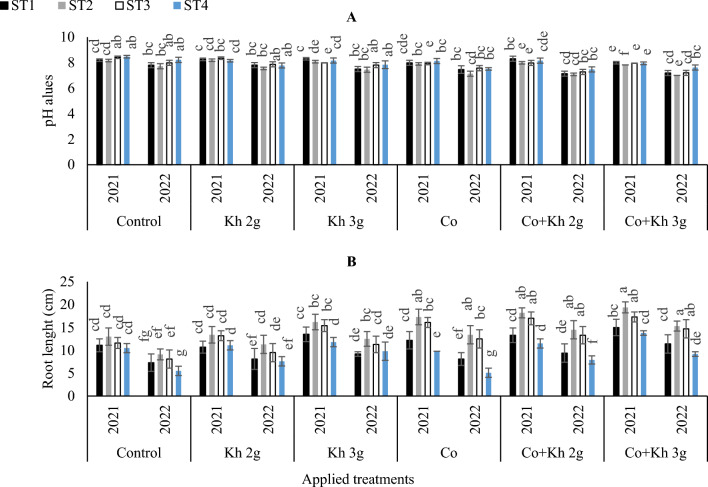
The impacts of implementing different water requirements strategies (STs) and applied cobalt applications combined with ground drench applications of Kh on the average (**A**) Soil pH values, (**B**) Root length at two seasons of 2021/2022. Data represent the mean of three replicates of both the years of 2021 and 2022. Error bars indicate standard errors from the mean. Bars with different letters are statistically significant at *p* ≤ 0.05. Abbreviations: Control, spray with pure water; Kh (applying soil application of potassium humate at two rates 2 and 3 g l^−1^); and Co (applied two equal doses of cobalt by (3.25 mg l^−1^ for the dose), injection in the irrigation water).

The maximum pH values were achieved through the adoption of ST3 and ST4 in the control treatment, in the first season. While in the second season, the maximum pH values were observed with the sole application of (Kh 2 g l^−1^ and Kh 3 g l^−1^) under ST3 and ST4 schemes. Although that significantly matched the adoption of control (ST3 and ST_4_). The lowest pH values were obtained through a combination of Kh 3 g l^−1^ applications adopting the ST2, in the first and second seasons (Fig. 8A). The highest root length was registered with the combined application of Co with (Kh 2 g l^−1^ and Kh 3 g l^−1^) under the (ST3 and ST4). However, that significantly matched the adoption of sole Co application under control ST2 in the two seasons (Fig. 8B). Whereas the lowest root length was obtained with the sole application of Co, in the first season, or by adopting either the sole application of Co and the control treatment under the ST4, in the second season.

#### Oil, protein and carbohydrate contents

**Figure 9:**
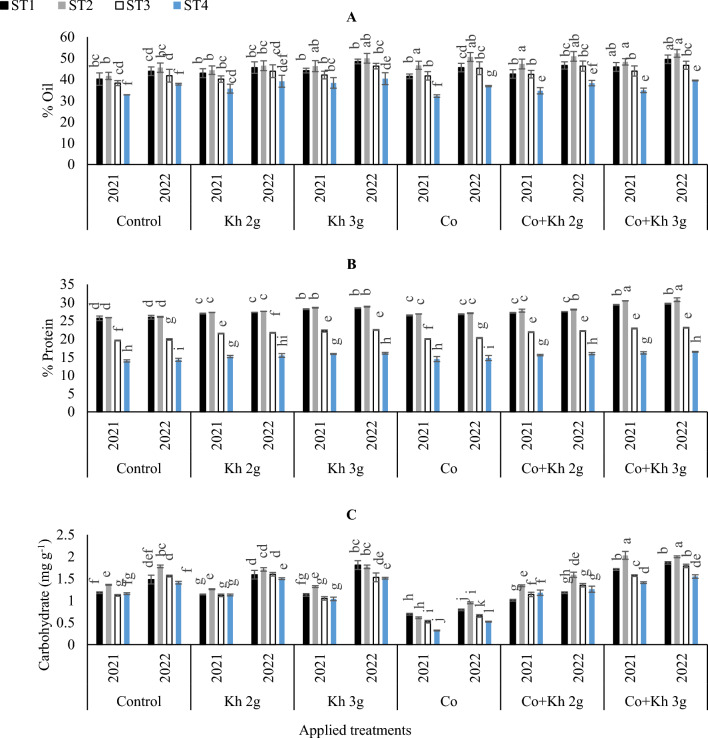
The impacts of implementing different water requirements strategies (STs) and applied cobalt applications combined with ground drench applications of Kh on the (**A**) Oil in seeds, (**B**) Protein, and (**C**) Carbohydrates in peanut seeds in two seasons of 2021/2022. Data represent the mean of three replicates of both the years of 2021 and 2022. Error bars indicate standard errors from the mean. Bars with different letters are statistically significant at *p* ≤ 0.05. Abbreviations: Control, spray with pure water; Kh (applying soil application of potassium humate at two rates 2 and 3 g l^−1^); and Co (applied two equal doses of cobalt by (3.25 mg l^−1^ for the dose), injection in the irrigation water).

Based on the data presented in (Fig. 9A), it can be observed that the oil content in the peanut seeds was higher under the (ST1 and ST2) compared to the (ST3 and ST4). The highest oil content was obtained when a combination of Co + Kh 3 g l^−1^ was applied with the (ST1 and ST2) schemes. However, that was significantly matched by applying Co × ST2 or Kh 3 g l^−1^ × ST2 or (Co + Kh 2 g l^−1^) × ST2 scheme, in both seasons. However, a significant reduction in oil content was observed in the control treatment where the ST4 scheme was adopted or when Co was applied under the ST4, in both seasons.

Similarly, protein content in peanut seeds was decreased when ST_3_ or ST_4_ schemes were implemented as depicted in (Fig. 9B). The lowest protein contents were obtained in the control treatment in conjunction with the S4, which was similar to adopting ST4 with the sole application of Co. The highest protein contents in peanut seeds were obtained with the adoption of (Co + Kh 3 g l^−1^) × ST2, in the first and second seasons.

On the other hand, the obtained results indicated that carbohydrate content attained higher increases in the second season than in the first. The maximum increase in the carbohydrate content was observed when a combined application of (Co + Kh 3 g l^−1^) was used along with ST2, in the first and second seasons. While the lowest carbohydrate content was registered with the sole application of Co under the ST4 (Fig. 9C).

#### Seed yield and WP

**Figure 10:**
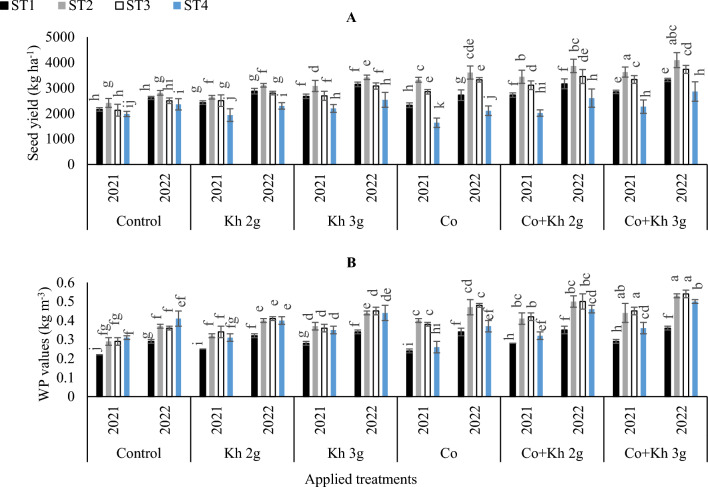
The impacts of implementing different water requirements strategies (STs) and applied cobalt applications combined with ground drench applications of Kh on the (**A**) Peanut seeds yield and (**B**) Water productivity (WP) in two seasons of 2021/2022. Data represent the mean of three replicates of both the years of 2021 and 2022. Error bars indicate standard errors from the mean. Bars with different letters are statistically significant at *p* ≤ 0.05. Abbreviations: Control, spray with pure water; Kh (applying soil application of potassium humate at two rates 2 and 3 g l^−1^); and Co (applied two equal doses of cobalt by (3.25 mg l^−1^ for the dose), injection in the irrigation water).

The data revealed that peanut production was significantly impacted by the various adopted ST schemes and the examined application treatments, as demonstrated in Tables 4 and 5**.** The combined application of (Co + Kh 3 g l^−1^) along with the adoption of the ST2 gave the highest value for seed yield, in the first and second seasons (Fig. 10A). While, the adoption of the ST4 along with the Co application resulted in a decreased yield, in the first and second seasons. Furthermore, the maximum WP was achieved by using Co + Kh 3 g l^−1^ along with adopting the ST2 and ST3, in both seasons. The minimum WP was obtained by adopting the ST1 in the control treatment, in the first and second seasons (Fig. 10B).

## Discussion

The appropriate nutritional and technical irrigation strategies under water stress conditions are considered effective tools to maintain the productivity of field crops to a large extent.

### Mechanisms by which plants deal with water stress and the role of Co and Kh under stress condition

A. Proline production

Based on the results obtained, it appears that the peanut yield was most negatively affected by the ST4 water stress scheme. The increase in the water stress under the ST4 resulted in the higher production of proline contents. Under stress conditions, peanut plants tend to activate the defense mechanisms such as increasing proline levels. Which perform multiple functions such as osmotic adjustment, maintaining turgor pressure and minimizing the harmful effects of water stress. The previous result aligns with the findings of^62–64^.

B. Regulation of proline, antioxidants and ROS

During stress conditions, plants tend to produce a higher amount of ROS which disrupts the metabolic functioning of plants^65^. Therefore, plants tend to accumulate osmolytes such as proline and carbohydrates in the cytoplasm. Where they act as molecular chaperones and help in maintaining protein structure and cell redox status^66^. However, under water scarcity, the water uptake and relative water content decrease leading to an increase in cytoplasmic viscosity. As a result, ROS production continues and causes significant damage, particularly in the biosynthesis of chloroplasts and mitochondria^65^. In this study, adopting the ST2 along with Kh alone or in combination with the Co maximally enhanced the antioxidants, which was aligned with the research by^67^. The enhanced content of antioxidants like SOD, POD, CAT and APX is due to the increased level of K^+^ ions and reduces the oxidative stress markers like H_2_O_2_ and proline and secondary metabolic compounds (phenolics) content under the water conditions.

### Effects of water stress on chlorophyll and RWC alongside the role of optimal ST, Co, and Kh

The reduction in water application resulted in a decreased amount of total chlorophyll content in the peanut plants. The ST2 along with the application of Kh 3 g + Co maximally improved the chlorophyll content. This increase in the chlorophyll content may be attributed to an increased level of K^+^ ions in the leaves which enhances photosynthesis and leads to higher carbohydrate synthesis. Cobalt applications might have improved the chlorophyll contents under various irrigation strategies, which is in line with previous research^17^. The co-application of Kh and Co maximally enhanced the RWC in peanuts under the ST2 water strategy. The enhancement was attributed to the role of Kh and Co in enhancing the tissue water status and membrane stability index while reducing the electrolyte leakage under water stress conditions. The previous findings corroborate with the results of^13,67–69^.

### Effects of Co and Kh under various STon nutrient homeostasis, pH, and root length

The mineral nutrient elements uptake like N, P, K, Ca, Fe, Mn and Zn were highly influenced by the different water strategies along with the application of Kh and Co. The co-application of Kh 3 g l^−1^ and Co enhances the macro and microelement uptake in plants by improving soil fertility and root growth and development. Moreover, the symbiotic associations with beneficial microbes play a crucial role in plants exploring a large volume of soil and accessing a greater pool of mineral nutrients. The results obtained from this study showed that previous research has highlighted the importance of Co applications for legume crops due to its key role in N-fixation^70–72^. However, the application of Co is correlated with the irrigation scheme used^14^. Specifically, the use of the ST4 led to the greatest reduction in yield attributes. This was attributed to changes in soil pH that occurred under stress schemes (ST3 and ST4) that led to a decline in nutrient availability and bio-exudate amounts from both the roots and microbiome in the rhizosphere^17,73,74^. These changes ultimately resulted in increases in soil pH and a decrease in peanut root length. In turn, this has led to a decrease in the availability of both macronutrients (N, P, K, and Ca) and micronutrients (Fe, Mn, and Zn) uptake. Furthermore, the current data indicated the antagonistic relationship between Co and Fe. In a similar context, studies have reported decreases in Fe uptake due to Co applications, although the mechanisms behind this have not yet been clearly defined^10,75,76^. However, Lwalaba et al.^77^ have suggested that these reductions are because Fe and Co share the same transporters. Our results confirm these observations and reveal high reductions in Fe accumulation in peanut leaves.

The soil pH decreased as the water requirement increased in the soil. The adoption of the ST4 showed the maximum soil pH value. The dearth of water in the soil would have resulted in a greater accumulation of undissolved alkaline salts on the soil surface which would have in turn increased the soil pH. These results are consistent with the previous studies of^78,79^. The increase in water stress would have resulted in a decrease in the root length of the peanut plant. The adoption of the Kh application appeared to confer substantial plant nutrient requirements for root membrane integrity and osmotic adjustment. The Kh also improves lateral root formation and root hair initiation in the test crop plant. Therefore, the Kh applications might have eventually led to improvements in peanut plant tolerance to water stress conditions. Our results are supported by the previous studies of^12,80–82^. However, the combined application of Co and Kh 3 g led to an increase in the root length under the adopted water strategies, especially in the ST2. The increase in the root length might be due to the prominent role of Kh 3 g and Co in regulating root growth and development in plants. These findings are in harmony with those of^83–85^.

### Hypotheses by which Co increases chlorophyll content and yield under water stress

One interesting finding in the current study was the pronounced improvements of chlorophyll content and yield by applying Co, especially under ST1 and ST2 schemes, although the observed reductions in Fe uptake. Such findings are significant as Fe plays a crucial role in the structure of chlorophyll and its biosynthesis^86–88^. This study assumed two hypotheses: (A) that may be attributed to the change in chlorophyll size due to Co molecules. This could be confirmed through microscopic analysis (which we didn't perform in this study). Consequently, this study predicts this modification in the chlorophyll cell size (which is smaller and more numerous). This led to more effective light-harvesting capacity and dry matter production through chlorophyll cells, reflected in improving peanut yield. In this regard^89,90^, demonstrated that smaller chlorophyll cells increase chlorophyll density per unit mass and produce more than 41% of oxygen per chlorophyll unit. While the other hypothesis (B) that absorbed Co molecules interfered with Fe due to their molecular similarity in size and charge^91^. That allowed it to interfere with the related functions of Fe including chloroplast formation. Previous studies, such as those by^92,93^, have reported similar findings for nutrient replacements under certain circumstances. For example, Na^+^ can replace K^+^ in some physiological processes without affecting yield and quality. Liu et al.^79^ also indicated that peanut plants showed high absorption activities of Na^+^ to replace the shortage in available K^+^ under salinity conditions. Thus, it was hypothesized that increased Co concentrations in the rhizosphere led to their entrance through either non-selective cation channels^94^ or transporters, which caused decreases in Fe uptake. Meanwhile, plants dealt with these amounts of Co by increasing water absorption, as a protected strategy. Hence in this study, notable increases in RWC were observed when Co was applied under different ST treatments. This is supported by previous studies indicating the vital role of Co play in water relations^10,14^ and RWC^12^. Nonetheless, some Co molecules interfered with Fe to complete chloroplast formation, as mentioned earlier, which explained the increases in chlorophyll contents. In a similar context, this finding is supported by^95,96^, who noticed a significant favourable impact of Co applications on chlorophyll contents, with the reduction of Fe uptake^10^. They found a strong association between Co and Fe oxides, resulting in the reduction of available Fe for plant uptake. Consequently, some Fe molecules became available within plants, although their total absorbed amounts were lower. At this point, it appears that plants redirected the available amounts of Fe toward increasing the production of antioxidants that Fe consider the main component, such as POD, CAT, and APX^97^. The improvements in POD, CAT and APX were pronounced especially under ST2 when Co was applied. That caused improvements in scavenging ROS components and maintained their concentrations within the safe limits. Therefore, photosynthesis continued, and yield was not affected under these conditions. However, both aforementioned hypotheses were in harmony with the obtained results. Further future studies are required to validate these hypotheses. Since deeper understanding of this issue will provide a basis for developing an appropriate irrigation strategy under Co utilization to improve the yield of peanuts in arid areas.

### Optimum treatment combination for peanut crop in arid conditions

The data obtained from the study showed that the combined application of Co and Kh 3 g under the ST2 resulted in the highest oil, protein, carbohydrate and seed yield. Whereas the adoption of the ST2 might provide the requisite amount of water required for the proper physiological functioning of the peanut plants. Moreover, this combination maximally enhanced the chlorophyll content, RWC, antioxidants, mineral elements and root length of the peanut plant. The increases in these parameters would have culminated in enhancing the overall performance of the peanut plants under the different water strategies. This in turn might have led to significant improvements in peanut yield and quality attributes especially under the ST2.

In this scenario, this study hypothesized that under ST2 peanut plants experienced a certain degree of water stress, which triggered a series of physiological effects. This leads peanut plants to rearrange their proline, antioxidant system, and nutrient accumulation to mitigate water stress. Under these circumstances, supplying stressed peanut plants with Co would allow them to improve nutrient uptake and also enhance antioxidant production. Regarding the related mechanisms of improving nutrient accumulation through the application of Co under ST2. Several researchers have noticed similar results; their explanations varied in specifying the decisive interpretation of this result. In this regard, according to the results obtained, the current study contributed mainly for three prominent reasons: **A:** Co role in enhancing water status within plant tissues by either increasing the uptake of the related nutrient with water relations such as (P, K, Zn, and Ca). The enhancement of K^+^ uptake in peanut leaves resulted in an improvement in the cell membrane permeability and RWC percentage. These results match with the findings of^98,99^. The increase in the uptake of Ca^2+^ under these conditions enhances plant cell membrane integrity and improves cellular signalling responses. While P enhances root development and architecture^100,101^. These enhancements in P uptake further contributed to improved carbohydrate synthesis, additionally, it led to raising the nutrient transport due to the decline in the osmotic potential of the sap^44,102^. The enhanced uptake of Zn^2+^ contents improves plant cell membrane stability, enhances the formation of chlorophyll, increases the osmolyte accumulation, and stomatal regulation, and increases the rate of photosynthesis^103,104^. **B:** Activated an effective and equilibrium antioxidant system. That involves the H_2_O_2_ and phenolics production, as well as the production of proline and antioxidant enzymes, these findings are in harmony with^105,106^. Where this balance is crucial for maintaining the integrity of cell membranes and alleviating the stress severity of Co and water reductions. Furthermore, that helps peanut plants execute several functions, including enhancing osmotic potential, preserving turgor pressure, and maintaining the integrity of the cell membranes and proteins^63,107^. **C:** On one hand, improving peanut root efficiency by enhancing root length and exudates, leading to decreased soil pH and enhancing the availability, acquisition and uptake of P. On the other hand, the application of Kh 3 g l^−1^ to the stressed peanut plants provided substantial amounts of plant nutrient requirements viz. K^+^ nutrient. This, in turn, has promoted water status and increased root secretions^108,109^ by decreasing the soil pH and allowing the nutrient availability and their uptake^81^. Moreover, Kh contributed by the chelating ability in formatting protective layers to maintain the micronutrients, especially Fe and Mn, ultimately causing some improvements in (Fe and Mn). Where the results in this study confirm these observations, these outcomes align with the findings of previous study^110^.

Therefore, the current study revealed that the combined application of Co and Kh 3 g l^−1^ under the ST2 resulted in the highest WP. The enhancement may be due to the improvements in the soil properties and enhancing the antioxidant defense system and the proline contents which has a substantial role in increasing the drought tolerance of the plants. These factors would have in turn enhanced the WP of the peanut plant.

## Conclusion

The results indicate that cobalt positively affected chlorophyll contents and peanut yield, despite an observed decrease in iron uptake. This contradicts the role that iron plays in the structure of chlorophyll. The assumption is that any decrease in iron accumulation leads to reduced chlorophyll, especially under stressful water conditions. The study suggested two hypotheses to explain this finding A) The modification in the chlorophyll cells size due to cobalt molecules B) cobalt-iron replacement in the chlorophyll structure. Nonetheless, the current study was unable to confirm these hypotheses due to limitations in the microscopic analysis under the current experimental conditions. Therefore, further research is needed to validate these hypotheses. The study found that the correlation between irrigation patterns and cobalt applications was inconsistent for chlorophyll content, antioxidant enzymes and nutrient accumulation. However, the maximum enhancement was registered in the ST2-adopted water scheme with the application of potassium humate and cobalt. Therefore, based on the findings, it is recommended to implement an ST2 irrigation scheme for the better production of peanuts. In addition, a combined application of cobalt and potassium humate at (3 g l^−1^) is recommended to improve macronutrient and micronutrient accumulation, chlorophyll content, relative water content, protein, oil, yield and water productivity of the peanut plant. This approach can benefit farmers or practitioners by boosting yield and WP of peanuts up to 62.0 and 53%, respectively. Which ultimately achieves the optimum conservation of irrigation and fertilization resources.

## Data Availability

All the presented datasets during this study are available from the corresponding author upon reasonable request.
